# Melanocortin Regulation of Inflammation

**DOI:** 10.3389/fendo.2019.00683

**Published:** 2019-10-09

**Authors:** Wei Wang, Dong-Yu Guo, Yue-Jun Lin, Ya-Xiong Tao

**Affiliations:** ^1^Department of Clinical Laboratory, Xiamen Huli Guoyu Clinic, Co., Ltd., Xiamen, China; ^2^Department of Anatomy, Physiology, and Pharmacology, College of Veterinary Medicine, Auburn University, Auburn, AL, United States

**Keywords:** melanocortin, melanocortin receptor, immune modulation, signaling, therapeutics

## Abstract

Adrenocorticotropic hormone (ACTH), and α-, β-, and γ-melanocyte-stimulating hormones (α-, β-, γ-MSH), collectively known as melanocortins, together with their receptors (melanocortin receptors), are components of an ancient modulatory system. The clinical use of ACTH in the treatment of rheumatoid arthritis started in 1949, originally thought that the anti-inflammatory action was through hypothalamus-pituitary-adrenal axis and glucocorticoid-dependent. Subsequent decades have witnessed extensive attempts in unraveling the physiology and pharmacology of the melanocortin system. It is now known that ACTH, together with α-, β-, and γ-MSHs, also possess glucocorticoid-independent anti-inflammatory and immunomodulatory effects by activating the melanocortin receptors expressed in the brain or peripheral immune cells. This review will briefly introduce the melanocortin system and highlight the action of melanocortins in the regulation of immune functions from *in vitro, in vivo*, preclinical, and clinical studies. The potential therapeutic use of melanocortins are also summarized.

## Introduction

The melanocortin system consists of the melanocortins, two endogenous antagonists, the agouti-signaling protein (agouti) and agouti-related peptide (AgRP), and five receptors. The melanocortins, including adrenocorticotropic hormone (ACTH), and α-, β-, and γ-melanocyte-stimulating hormones (α-, β-, γ-MSHs), are derived from post-translational processing of a common precursor pro-opiomelanocortin (POMC). In addition to the well-documented adrenal responses stimulated by ACTH and pigmentary effects induced by α-MSH, other physiological functions, including energy homeostasis, sexual activity, exocrine secretion, as well as anti-inflammatory and immunomodulatory actions, exerted by these versatile neuropeptides upon the host, have also been reported extensively (1–3).

The first clinical experiment applying ACTH in the treatment of patients with severe rheumatoid arthritis, rheumatic fever, and certain other conditions, was performed by Hench et al. (4, 5), the Nobel Laureates in Physiology or Medicine in 1950 for their milestone discoveries on ACTH and the adrenal hormone cortisol (6). At that time, ACTH-induced improvement in clinical features in rheumatoid arthritis patients was thought to be via stimulation of hypothalamus-pituitary-adrenal gland axis and production of glucocorticoids.

Our understanding of melanocortin physiology and pharmacology has greatly improved with the recognition and cloning of melanocortin receptors (MCRs). Indeed, it was not until 2002 that local activation of melanocortin-3 receptor (MC3R) by ACTH, independent of glucocorticoid, was demonstrated to be also responsible for ACTH efficacy in gouty arthritis (7). This important study suggested that selective MC3R agonists might be utilized as novel anti-inflammatory agents for clinical management of chronic inflammatory conditions (7). In this review, we will briefly introduce the melanocortin system, including melanocortin peptides, MCRs, and intracellular signaling pathways. Then, the anti-inflammatory effects of melanocortins *in vitro* and *in vivo*, the underlying molecular mechanisms, as well as potential therapeutic use of melanocortins, will be elaborated.

## The Melanocortin System

### The Melanocortin Ligands

The melanocortins are derived from post-translational processing of a common precursor protein, POMC, first discovered in mouse pituitary tumor cells and later demonstrated in human nonpituitary tumor cells by several independent groups (8–10). Using recombinant DNA technology, Nakanishi et al. first reported the nucleotide sequence of the cDNA for bovine POMC (11). Subsequently, POMC sequences were reported in several mammals, amphibians, and teleosts (12–16). Sequence comparison revealed a recognizable POMC sequence in lamprey, the most ancient vertebrate, that shares structural similarity to those of higher vertebrates and teleosts, indicating the appearance of POMC might date back to 700 million or more years (17).

In addition to the pituitary and melanocytes and keratinocytes in skin where POMC were originally discovered, POMC mRNA has been found in hypothalamic arcuate nucleus, nucleus of solitary tract in the caudal brainstem, spinal cord, and dorsal root ganglion (18–21). POMC mRNA is also detected in peripheral immune cells such as lymphocytes and monocytes, suggesting a regulatory role of POMC-derived peptides in inflammation in these immune-related cells (22–26). Indeed, cytokine-, interferon-, or hormone-induced activation of signal transducers and activators of transcription signaling cascade enhances POMC expression and melanocortin synthesis at sites of infection or inflammation [reviewed in (27)].

The tissue-specific post-translational processing of POMC is performed by prohormone convertases (PCs), proteolytic enzymes belonging to the family of serine-type proteinases. PC1 cleaves POMC to produce ACTH and other peptides in the anterior pituitary gland, whereas in lower vertebrates and during fetal and infantile periods in humans, PC2 located in the pars intermedia accounts for the production of MSHs and β-endorphin. In addition, POMC is further processed into MSHs by PC2 in peripheral tissues such as skin and hair follicles and in central nervous system (CNS) (28–30).

All melanocortins share the common amino acid motif HFRW, which is the minimum sequence required for receptor binding and activation. It should be noted that each melanocortin exerts different affinities for the specific receptor: ACTH is specific for the activation of MC2R, α-MSH exhibits the highest affinity for MC1R, MC4R, and MC5R, whereas γ-MSH displays the highest affinity for MC3R (as shown in Table 1). The melanocortin system is unique due to the existence of two natural endogenous antagonists, agouti, and AgRP, which act as inverse agonists to block the agonist binding at the MCRs and to decrease the constitutive activity (agouti for MC1R and AgRP for MC3R and MC4R). Recently, AgRP, through activating neural MC4R, was shown to induce Gi protein activation and potassium channel opening in a Gs-independent manner (53, 54). We also showed that AgRP decreases cAMP activity of constitutively active F347A hMC3R but stimulates ERK1/2 activation in both wild type and laboratory-generated mutant F347A hMC3Rs (55, 56). In the MC4R, AgRP is also an inverse agonist at the Gs-cAMP pathway but an agonist at the ERK1/2 pathway (57). These data demonstrated biased agonism of AgRP in neural MCRs, adding a novel layer of complexity to MCR signaling (58, 59).

**Table 1:** The pharmacology of the MCRs.

**MCR**	**Ligand**	**Modified from^d^**	**Biological effects**	**Tested models**	**References**
MC1R	α-MSH = ACTH > β-MSH > γ-MSH^a^	Endogenous ligands	Induces skin pigmentation, anti-inflammatory	Rodents	(2, 31, 32)
	BMS-470539^a^	Novel small molecule	Anti-inflammatory	LPS-induced lung inflammation, PAF-induced vascular inflammation	(33, 34)
	AP1189^a^	Novel small molecule	Anti-inflammatory	Zymosan-induced peritonitis, K/BxN serum-induced arthritis	(35)
	Agouti^b^	Endogenous ligand	Inhibits eumelanin production	Lethal yellow and variable yellow alleles (A^y^ and A^vy^) mice	(36)
MC2R	ACTH^a^	Endogenous ligand	Induces steroidogenesis, anti-inflammatory	Rodents	(2, 31, 32)
MC3R	γ-MSH ≥ ACTH = β-MSH = α-MSH^a^	Endogenous ligands	Energy homeostasis, cardiovascular function	Rodents	(2, 31, 32)
	MTII^a^	α-MSH	Inhibits neutrophil migration and cytokine generation	Crystal-induced peritonitis	(37)
	D-Trp^8^-γMSH^a^	γ-MSH	Anti-inflammatory, stimulates food intake	Crystal-induced inflammation, ischemia-reperfusion injury, and inflammatory arthritis;	(38–42)
	AP214^a^	α-MSH	Anti-inflammatory	Zymosan-induced peritonitis, inflammatory arthritis	(43)
	AP1189^c^	Novel small molecule	Anti-inflammatory, promotes resolution of acute inflammation	Zymosan-induced peritonitis, K/BxN serum -induced arthritis	(35)
	SHU-9119^b^	α-MSH	Inhibits D-Trp^8^-γMSH-induced anti-inflammatory effects	Crystal-induced peritonitis	(37)
	AVM-127^b^	γ-MSH	inhibits α-MSH-induced pro-erection	Male rats	(44)
MC4R	α-MSH = ACTH > β-MSH > γ-MSH^a^	Endogenous ligands	Energy homeostasis, erectile function, cardiovascular function	Rodents	(2, 31, 32)
	THIQ^a^	Novel small molecule	Anti-inflammatory, inhibits food intake, promotes penile erection	LPS-induced brain inflammation, male rat ex copula	(45, 46)
	Ro27-3225^a^	α-MSH	Inhibits 4 h food intake	Rats	(47)
	PT-141^a^	MTII	Induces transient erections	Male Sprague-Dawley rat and men	(48, 49)
	AgRP^b^	Endogenous ligands	Increases food intake, decreases mean arterial pressure and heart rate	Lethal yellow(A^y^), variable yellow alleles(A^vy^), obese (ob/ob), and diabetic (db/db) mice	(2, 31, 32)
	ML00253764^b^	Novel small molecule	Reduces tumor-induced weight loss	CT26 colorectal tumor and Lewis lung carcinoma tumors	(50, 51)
	Ro27-4680^b^	α-MSH	Increases acute food intake	Rats	(47)
MC5R	α-MSH > ACTH > β-MSH > γ-MSH^a^	Endogenous ligands	Exocrine function, anti-inflammatory	Rodents	(2, 31, 32)
	PG-901^a^	SHU-9119	Anti-hypertrophic, reduces cellular glucose uptake	STZ-diabetic Sprague Dawley rats	(52)

a indicates agonist;

b indicates antagonist;

c indicates biased agonist;

d *indicates which template drug the corresponding ligand is derived from*.

### The Melanocortin Receptors

The versatile actions of melanocortin peptides are mediated by five MCRs, MC1R to MC5R, which are named according to the sequence of their cloning. As members of Family A G protein-coupled receptors, MCRs share the common hallmark structure consisting of seven α-helical transmembrane domains, an extracellular N terminus and intracellular C tail, connected by three alternating extracellular and three intracellular loops. Sequence comparison of human MCRs reveals high homologies, from 38% identity between MC2R and MC4R to 60% identity between MC4R and MC5R (27). As shown in Table 2, the MCR exerts diverse expression patterns, which might contribute to their multifaceted physiological functions. As challenging and popular drug targets, the MCRs have triggered intensive efforts to develop more potent and selective melanocortin agonists and antagonists. Table 1 includes some of the most commonly-used peptide and small molecule ligands for the MCRs.

**Table 2:** The distribution of the MCRs.

**MCR**	**mRNA expression**	**Techniques**	**Species^a^**	**References**
MC1R	Present in skin and melanocytes; Absent in thyroid and testis	Northern blotting	Human (Cloudman S91 or WM264-1 melanoma cells)	(60, 61)
	Present in periaqueductal gray matter; Absent in amygdala, septal area, thalamus, midbrain, and hypothalamus	*In situ* hybridization	Rat	(62)
	Present in macrophages, monocytes, lymphocytes, neutrophils, and astrocytes	RT-PCR^b^, S1 nuclease assays, and degenerate PCR	Murine (RAW264.7), human (THP-1 or A172 cells, neutrophils, or peripheral blood mononuclear cells)	(63–67)
MC2R	Present in zona fasciculata and zona glomerulosa of adrenal gland; Absent in medulla and capsule of adrenal gland, pituitary, liver, lung, thyroid, and kidney	*In situ* hybridization and Northern blotting	Rhesus macaque	(61)
	Present in chondrocytes and osteoblasts	RT-PCR^b^	Human (primary articular chondrocytes or osteoblasts)	(68, 69)
MC3R	Present in brain and placenta; Absent in adrenal gland, pancreas, stomach, kidney, spleen, liver, and lung	Northern blotting	Rat or canine	(70, 71)
	Present in cortex, thalamus, hypothalamus, hippocampus, amygdala, and septum	*In situ* hybridization	Rat or mice	(70, 71)
	Present in monocytes and macrophages	RT-PCR^b^ and Southern blotting	Mouse (peritoneal macrophages) or human (THP-1 cells)	(64, 72)
MC4R	Present in brain; Absent in heart, ovary, testis, spleen, kidney, lung, stomach, colon, pancreas, jejunum, cortex, cerebellum, salivary gland, liver, adrenal,	Northern blotting	Canine or rat	(73, 74)
	Present in thalamus, hypothalamus, dentate gyrus, cortex, amygdala, and CA1 and CA2 regions of hippocampus; Absent in CA3 and CA4 regions of hippocampus.	*In situ* hybridization	Mouse	(73)
	Present in thalamus, hypothalamus, brainstem, spinal cord, olfactory cortex, hippocampal formation, amygdala, septal region, corpus striatum, bed nucleus of the stria terminalis, midbrain, pons, and medulla oblongata.	*In situ* hybridization	Rat	(74, 75)
MC5R	Present in skeletal muscle, lung, spleen, brain, Absent in melanoma cells, adrenal tissues, and placenta	Northern blotting	Rat, mouse or human (A375 melanoma cells)	(76)
	Present in skin, skeletal muscle, thymus, bone marrow, adrenal gland, testis, ovary, and uterus; Absent in lung, kidney, esophagus, stomach, duodenum, jejunum, ileum, colon, liver, pancreas, prostate, seminal vesicle, spinal cord, dorsal root ganglion, sciatic nerve and soleus muscle	RNase protection assays	Mouse	(19, 77)
	B lymphocytes	RT-PCR^b^	Mouse (Ba/F3 lymphocytes)	(78)
	Splenic lymphocytes	Radioligand binding assay	Rat (mononuclear lymphocytes)	(79)
	T lymphocytes	Immunohistochemistry	Mouse (CD25+CD4+ regulatory T cells)	(80)

a indicates the species from which the tissues or cell types are collected;

b *reverse transcription-PCR*.

The MC1R is the classical MSH receptor expressed in skin and hair follicles that regulates pigmentation. However, MC1R, with the highest affinity for α-MSH, is expressed in virtually all cell types responsive to the anti-inflammatory action of melanocortins. It was also shown that normal human monocytes and mouse brain contain a low number of MC1R binding sites which is upregulated by various stimuli such as lipopolysaccharide (LPS) or combinations of cytokines, and traumatic brain injury, respectively (81, 82). In addition, by measuring ionized calcium binding adaptor molecule-1 staining (Iba-1) in microglial cells, a known marker for cerebral inflammation, Schaible et al. showed that α-MSH (11–13), probably by targeting the MC1R, significantly attenuates cerebral inflammation, as evidenced by reduced activation of Iba-1 positive cells in the ipsilateral hemisphere (82). Two synthetic MC1R agonists, BMS-470539 and AP1189, were also reported to exhibit anti-inflammatory activities in various inflammation models (33–35).

The MC2R, the classical ACTH receptor, is expressed in the adrenal cortex that regulates adrenal steroidogenesis and cell proliferation (Table 2). MC2R is also expressed in chondrocytes and osteoblasts, where it might be involved in the regulation of local inflammation (68, 69).

As shown in Table 2, the neural MCRs, MC3R, and MC4R, are expressed primarily in the CNS and regulate energy homeostasis (83–85). However, the MC3R is also expressed in human placenta, heart, and gut as well as lymphocytes and macrophages, where it mediates potent anti-inflammatory effects of MC3R agonists such as MTII, D-Trp^8^-γMSH, AP214, and AP1189 (35, 37–39, 43, 70, 86, 87), whereas MC3R antagonists, SHU-9119 and AVM-217, prevents such protective effects (37, 44).

Extensive localization studies on rodent brains showed that the *Mc4r* mRNA is expressed in multiple brain regions, including cortex, brainstem, thalamus, hypothalamus, hippocampus, and spinal cord (73–75). The MC4R has been reported to mediate central anti-inflammatory effects of α-MSH. Schaible et al. showed that MC4R-mediated activation of anti-apoptotic pathways might at least in part explain the neuroprotective properties of α-MSH in a mouse model of traumatic brain injury (82), which is similar to the actions of MC4R ligands observed in animal models of cerebral ischemia (88–90).

The MC5R is widely expressed in the skin, adrenal glands, adipocytes, skeletal muscle, bone marrow, kidney, liver, lungs, spleen, thymus, gonads, uterus, and brain (76, 77, 91–93). *Mc5r* knockout mice display a phenotype with defective water repulsion and thermoregulation due to decreased sebogenesis, indicating a role for the receptor in sebum synthesis and thermoregulation (93). Additional studies suggested that the receptor also contributes to the immunomodulatory functions in B and T lymphocytes, and mast cells (78, 80).

### Intracellular Signaling Pathways

It is accepted now that, once activated by the agonists, the MCRs at the cell surface undergo conformational changes and trigger a complex of intracellular network of pathways. The MCRs are primarily coupled to Gs proteins and result in activation of adenylyl cyclase, an enzyme that catalyzes the conversion of cytoplasmic ATP to cAMP. As a second messenger, cAMP activates protein kinase A, which further induces the phosphorylation of transcription factor, cAMP response element-binding protein, and affects the transcription of downstream genes.

In addition to this canonical cascade, MCRs can also signal through other pathways. For instance, all MCRs have been reported to induce ERK1/2 phosphorylation and intracellular Ca^2+^ mobilization, although the specific underlying mechanisms are not exactly the same with all five MCRs (94–99). The Gi protein was shown to be associated with MC3-5R activation and Gq was indicated to couple with MC4R (53, 96, 98, 100). In addition, MC1R and MC2R were shown to signal through p38 pathway in human HaCaT keratinocytes upon stimulation of ACTH (101). Phosphorylation of Janus kinase/signal transducer and activator of transcription in Ba/F3 cells and human cultured IM-9 lymphocytes expressing MC5R (78, 102) and c-Jun N-terminal kinase in HEK293 cells overexpressing MC4R were also reported (78, 102). However, some of these non-canonical signaling needs to be independently confirmed by different groups.

## ACTH and Inflammation

### ACTH Effects *in vitro*

The fact that ACTH is superior to corticosteroids in treating certain inflammatory diseases has raised a possibility that ACTH might have some therapeutic effects other than stimulating the levels of endogenous cortisol. In normal human keratinocytes, ACTH_1−39_ was shown to suppress NFκB activation stimulated by tumor necrosis factor-α (TNF-α), likely via enhancing nuclear translocation of the NFκB inhibitor IκBα to the nucleus (103). Further immunofluorescent labeling and western blot studies detected MC1R and MC2R in normal human keratinocytes, which might mediate the peptide-induced suppression on activation of NFκB (103).

In addition, the ability of ACTH to directly modulate local CNS inflammation, a factor in, and perhaps initiates, many CNS diseases, has been reported in several *in vitro* studies. Using rat brain cultures containing oligodendroglia, astrocytes, and microglia preincubated with cytotoxic agents, ACTH_1−39_ was shown to protect mature oligodendroglia and oligodendroglia progenitor cell (OPC) from death induced by staurosporine, AMPA, NMDA, kainate, quinolinic acid, or reactive oxygen species (ROS) (104, 105). In their studies, preincubation of the cytotoxic agents caused 50–75% death of mature oligodendroglia but little or no death of astrocytes or microglia (104). Since oligodendroglia and OPC uniquely provide metabolic support to neurons/axons, the protection of oligodendroglia and OPC from several excitotoxic and inflammation-related insults is of great importance in keeping the integrity of CNS (104).

Using rat glial cultures, the same group demonstrated that ACTH_1−39_ induces proliferation of OPC and accelerates differentiation of platelet-derived growth factor receptor-α (a phenotypic marker for OPC) positive OPC to a later stage characterized by greater expansion of oligodendroglia myelin-like sheets compared to untreated cells (105). Adenylyl cyclase was suggested to be involved in ACTH-mediated regulation of OPC proliferation, differentiation, and anti-inflammatory actions based on the findings that (1) OPC have delayed maturation and accelerated proliferation when adenylyl cyclase is activated *in vitro* (106, 107), (2) pituitary adenylyl cyclase-activating polypeptide-deficient mice have earlier onset of myelination, less time for axonal development, and synapse formation, as well as less neuronal plasticity (108), and (3) cAMP-inducing agents prevent oligodendroglial excitotoxicity and protect mature oligodendroglia from excitotoxic agents (109).

Furthermore, the same group showed that ACTH also protects rat forebrain neurons, the most vulnerable cells in CNS, from apoptotic, excitotoxic, and inflammation-related damage in a similar manner as mature oligodendroglia and OPC (110). Since excitotoxic damage to neurons is an important cause to several experimental and clinical CNS diseases, it is reasonable to explain the therapeutic benefits of ACTH in several inflammatory animal models of CNS disorders (110). However, the contribution from direct effects on oligodendroglia, OPC, or neurons within the brain and whether such beneficial actions can be observed *in vivo* in human patients remain to be investigated.

### ACTH Effects *in vivo*

The efficacy of treatment with ACTH in human and animal rheumatoid arthritis and gouty have been demonstrated since 1940s. In a murine model with monosodium urate (MSU) crystal-induced gouty arthritis, Getting et al. demonstrated that ACTH_4−10_ (MEHFRWG), lacking any glucocorticoid stimulating action, inhibits macrophage activation as evidenced by reduced phagocytosis and keratinocyte-derived chemokine (KC) release, and neutrophil accumulation as evidenced by decreased IL-1β (a pro-inflammatory cytokine) release in the inflammatory exudates (72). Since only *Mc3r* mRNA is detected in mouse peritoneal macrophages by RT-PCR, they proposed that ACTH_4−10_ attenuates KC release and possibly synthesis of other cytokines, and subsequent reduction of the host inflammatory response by targeting the MC3R expressed in macrophages (72). Similarly, in a rat model of arthritis, the same group showed that local injection of ACTH_1−39_ have significant anti-inflammatory effects, with inhibition of 82, 88, and 75% on neutrophil influx, joint swelling, and arthritis score, respectively (7). Comparable degrees of attenuation in the synthesis and/or release of the cytokines IL-1β and IL-6 were also observed (7). Interestingly, the anti-inflammatory actions of ACTH_1−39_ retain in adrenalectomized rats (7). Together with the observations that MC3R/MC4R antagonist, SHU9119, blocks ACTH anti-inflammatory actions and a selective MC3R agonist, γ_2_-MSH, retains the anti-inflammatory activity, it is reasonable to propose that partial anti-inflammatory effects of ACTH might be achieved by targeting MC3R expressed in rat knee joint macrophages (7).

Furthermore, ACTH was also reported to reduce fever after peripheral administration (5). Subsequently, Kass et al. demonstrated that intramuscular injection of ACTH significantly reduces leukocytic pyrogen-induced fever in human and rabbits (111). Together with the evidence that intracerebroventricular administration of ACTH_1−24_ also produces antipyresis (112), it can be hypothesized that the antipyretic effects of ACTH might be achieved through altering the activity of hypothalamic heat-regulating centers. However, further findings that central and peripheral injections of ACTH_1−24_ reduce fever in adrenalectomized rabbits exclude the necessity of corticosteroid participation in ACTH-induced fever reduction. This is of great importance considering that ACTH-induced corticosteroids readily cross the blood-brain barrier and can reduce fever when administrated in intrahypothalamic manner (113, 114).

### Mechanism of Anti-inflammatory Effects of ACTH

It is well-known that ACTH-mediated anti-inflammatory effects can be achieved through glucocorticoid-dependent and -independent manners. Regarding glucocorticoid-dependent action, ACTH is unique among melanocortins since it is the only peptide that activates MC2R. Generally, stimuli such as stress or inflammation trigger the synthesis and release of corticotropin-releasing hormone from the paraventricular nucleus of the hypothalamus, which further stimulates the release of ACTH from the pituitary. ACTH then circulates to the adrenal gland, where it activates MC2R located on the adrenal cortex and induces rapid synthesis of cortisol. Cortisol then activates glucocorticoid receptor and triggers downstream anti-inflammatory actions through genomic and non-genomic pathways, eventually resulting in decreased production of cytokines, chemokines, and inducible NO synthase, increased anti-inflammatory mediators, and phagocytosis of apoptotic neutrophils [reviewed in (6)]. It is also reported that glucocorticoids produced in the skin can also modulate local inflammation, although the underlying mechanism is not clear (115).

It is not until 2002, 50 years after the approval of ACTH for the treatment of many inflammatory conditions, that Getting et al. revealed the retention of anti-inflammatory effects of ACTH in adrenalectomized rats with knee gout, suggesting the existence of hypothalamus-pituitary-adrenal- and glucocorticoid-independent anti-inflammation mechanism (7). Until now, extensive *in vitro* and *in vivo* studies, as discussed above, have undoubtedly supported the notion that ACTH can exert its anti-inflammatory effects directly through activation of certain MCRs expressed in peripheral immune cells and hypothalamic neural cells.

### Therapeutic Use of ACTH in Inflammatory Diseases

Currently, the practical therapeutic use of ACTH or its analogs are merely based on its ability to induce glucocorticoid synthesis and release. Two commercialized ACTH formulations are available in the market in the United States. One is the repository corticotropin injection (RCI), a highly purified porcine ACTH analog approved by the FDA for the treatment of many autoimmune and inflammatory diseases since 1952. Currently, RCI is considered first-line therapy for infantile spasms. For other autoimmune disorders such as rheumatoid arthritis, multiple sclerosis relapses, symptomatic sarcoidosis, systemic lupus erythematosus, proteinuria in nephrotic syndrome, and dermatomyositis/polymyositis, it is utilized primarily as later-line therapy in patients whose conditions exacerbate, or adjunctive option in patients fail to respond to or cannot tolerate the conventional therapies (116).

The initial idea of utilizing ACTH as a therapeutic drug in the treatment of inflammatory conditions such as rheumatoid arthritis was proposed by Hench et al. (4). However, the emerging of glucocorticoid therapy makes ACTH the second choice or an adjunctive therapy to combat the inflammation associated with rheumatoid arthritis. Indeed, in two independent prospective clinical trials attempting to assess the efficacy and safety of RCI as adjunctive therapy in rheumatoid arthritis patients, Gaylis et al. showed that eight of ten patients received methotrexate plus RCI have improved clinical symptoms as supported by increased Clinical Disease Activity Index scores. Two patients have disease remission and three patients show low disease activity (117). Similarly, Gillis et al. demonstrated that, after subcutaneous injection with RCI over 12 weeks, all six patients with refractory rheumatoid arthritis achieve significantly lower Disease Activity Score, decreased tender and swollen joint counts, and reduced physician global visual analog scale (118). In addition, significant improvement were also observed in terms of Health Assessment Questionnaire score (3/6), erythrocyte sedimentation rate (4/6), and C-reactive protein levels (4/6) (118).

In several clinical studies aiming to compare the efficacy of RCI and other conventional therapies for infantile spasms treatment, Baram et al. evaluated the efficacy of RCI and prednisone in 29 enrolled infants, and they showed that 86.6% of RCI-treated infants and 28.6% of prednisone-treated infants have cessation of spasms and elimination of hypsarrhythmia (119). Similar results were obtained by Knupp and colleagues, who performed a prospective, observational, multicenter study to compare the efficacy of RCI, prednisolone, vigabatrin, and other non-standard therapies in 230 enrolled infants diagnosed with infantile spasms. At 2 weeks of treatment, 68% of RCI-treated infants have evident response, compared with 49% for vigabatrin and 22% for non-standard medications (120). At 3 months of treatment, the response rate of RCI-treated infants is 55%, significantly higher than those treated with prednisolone (39%), vigabatrin (36%), and non-standard medications (9%) (120). Those results convincingly indicated that, RCI therapy is more effective than other standard therapies in the treatment of infantile spasms. Therefore, the American Academy of Neurology and Child Neurology Society recommended, in the 2004 infantile spasms guideline, that ACTH can be considered as short-term treatment of infantile spasms (level B evidence).

Several studies also pointed to the possibility of RCI therapy as an alternative option for the treatment of multiple sclerosis relapse. In a randomized, double-blind, placebo-controlled, multi-site trial consisting of 197 patients, the Disability Status Scale, known as the gold standard of outcome parameter in multiple sclerosis relapse trials, was monitored weekly for 4 weeks. Rose et al. showed that 65% RCI-treated patients have improved Disability Status Scale compared to 48% of those treated with placebo gel. The response rates to RCI therapy are 43 and 30% higher than those to placebo treatment at week 1 and week 3, respectively (121, 122). In a prospective, randomized, open-labeled pilot trial, Simsarian et al. showed that intramuscular and subcutaneous administration of RCI for 5 days causes comparable alleviation in symptoms (123).

In the past several decades, more than two dozen clinical and healthcare utilization studies of RCI in the treatment of autoimmune and inflammatory disorders have been performed, and the retrospective analyses convincedly suggested that initiation of RCI therapy in patients with autoimmune diseases reduces post-therapy healthcare utilization such as hospitalizations, hospital length of stay, outpatient visits, and emergency department visits, therefore improves patient quality of life and mitigates the economic burdens on families, healthcare systems, and society (116).

The second formulation, cosyntropin, a synthetic analog composed of the first 24 amino acids of ACTH (ACTH_1−24_), is as potent as the full-length peptide in terms of steroidogenic activity. However, cosyntropin used in the ACTH stimulation test is only intended for the diagnose of adrenal insufficiency in the USA. In the UK, ACTH_1−24_, labeled as Synacthen Depot®, in addition to diagnostic use, is utilized as an adjunctive and short-term option for the glucocorticoid therapy in conditions when patients fail to respond or cannot tolerate glucocorticoid treatment (6).

## **α**-MSH and Inflammation

### **α**-MSH Effects *in vitro*

The molecular mechanisms of α-MSH-mediated anti-inflammatory functions have been studied extensively *in vitro* (summarized in Table 3). α-MSH-mediated inhibition of NF-κB activation, a well-known master regulator of inflammation that controls the expression of many cytokines, cytokine receptors, chemokines, growth factors, and adhesion molecules, was first discovered by Manna and Aggarwal, who demonstrated that α-MSH completely abolishes TNF-α-induced NF-κB phosphorylation in a dose- and time-dependent manner (124). It also blocks LPS-, okadaic acid-, and ceramide-mediated activation of NF-κB in human monocytic, epithelial, glioma, and lymphoid cells (124). Subsequently, similar effects exerted by α-MSH were reported in a variety of cell types including human microvascular endothelial cells (125), human melanocytes and melanoma cells (126), human glioma cells (127, 128), human pulmonary epithelial cells (129), human keratinocytes (103), mast cells (130), Schwann cells (131), human macrophages and neutrophils (132), human dermal fibroblast cells (133), and rat small intestine cells (152). Suppression of NF-κB translocation is achieved through generation of cAMP, activation of protein kinase A, and protection of IκBα from phosphorylation (124). Recently, α-MSH was reported to attenuate TNF-α-induced matrix metalloproteinase-13 expression by regulating activation of p38 and NF-κB in HTB-94 cells (a human chondrosarcoma cell line expressing MC1R), indicating that α-MSH might be used to prevent matrix metalloproteinase-13-mediated collagen degradation (153).

**Table 3:** Molecular mechanisms of α-MSH-mediated anti-inflammatory functions.

**Actions of α-MSH**	**Effectors**	**Cell types investigated**	**References**
Inhibition of transcription factor	NF-κB	Human monocytic U937, epithelial HeLa, glioma H4, lymphoid Jurkat cells, mast cells, Schwann cells, microvascular endothelial cells, dermal fibroblast cells, melanocytes and melanoma cells, keratinocytes, macrophages/neutrophils, and rat small intestine cells	(124–133)
Suppression of proinflammatory cytokines	TNF-α	Human A-172 and THP-1 cells, murine fibroblast L292 cells, and microglial N9 cells	(63, 64, 134, 135)
	IL-1, IL-6, and IL8	Rat PBMCs, murine peritoneal macrophages, murine microglial N9 cells, HaCaT cells	(7, 134, 136, 137)
	Groα	HaCaT cells	(138)
	KC	Murine peritoneal macrophages	(139)
Suppression of non-cytokine proinflammatory mediators	NO	RAW 264.7 cells, THP-1 cells, human melanoma FM55 cells, murine microglial N9 cells, rat PBMCs and astrocytes	(65, 134, 135, 140–143)
	PGE_2_	Human melanoma FM55 and HaCaT cells, and rat astrocytes	(142, 144)
	ROS	Rat peritoneal neutrophils	(145)
Suppression of adhesion molecules	ICAM-1	Human normal and malignant cells, dermal papilla cells and fibroblasts, and murine mast cells	(130, 133, 146, 147)
	CD40 and CD86	Human monocytes and peripheral blood-derived dendritic cells	(81, 148)
	VCAM-1	HMEC-1 and HDMECs	(125, 149)
	E-selectin	HMEC-1 and HDMECs	(125, 149)
Induction of cytokine suppression	IL-10	Human monocytes	(150, 151)

Extensive studies have demonstrated that α-MSH exerts the anti-inflammatory actions by suppressing the expression of proinflammatory cytokines such as TNF-α, interferon-γ (IFN-γ), IL-1, IL-6, IL-8, and KC. Lipton and colleagues showed that α-MSH, presumably by acting through MC1R, inhibits bacterial endotoxin-induced TNF-α production in human glioma cells (63). Similar inhibitory effects on TNF-α production exerted by α-MSH were observed in human monocyte/macrophages (64), melanocyte and melanoma cells (126), and human keratinocytes (103). Taylor et al. demonstrated that constitutive picomolar α-MSH detected in normal aqueous humor of human, rabbits, and mice is able to suppress the production of antigen-stimulated IFN-γ (154, 155). In addition, after preincubation of human peripheral blood mononuclear cells (PBMCs) with mitogen and different concentrations of α-MSH, Luger et al. showed that, in human PBMCs, the mitogen-induced transcription and biological activity of IFN-γ are significantly blocked by α-MSH in a dose-dependent manner, as evidenced by changes in IFN-γ mRNA expression and the major histocompatibility complex class I antigen expression, respectively (156). The production of other proinflammatory cytokines, including IL-1, IL-6, IL-8, Groα, and KC, when co-incubated with or without proinflammatory stimuli, are also inhibited by α-MSH (7, 136–138).

The ability of α-MSH in suppressing TNF-α-, IFN-γ-, or LPS-induced expression of intercellular adhesion molecule-1 (ICAM-1) have been reported in human normal and malignant cells, human dermal papilla cells and fibroblasts, and murine mast cells (130, 133, 146, 147). The inhibition of LPS-induced vascular cell adhesion molecule-1 (VCAM-1) and E-selectin expression were also reported in human microvascular endothelial cells (HMEC-1) and human dermal microvascular endothelial cells (HDMECs) (125, 149). In addition, α-MSH also regulates the expression of CD86 and CD40, cell surface molecules required for antigen presentation in monocytes and dendritic cells. Indeed, α-MSH was shown to, likely by acting at MC1R, down-regulate the surface expression of CD86 in both LPS-treated human monocyte and non-stimulated human peripheral blood-derived dendritic cells (81, 148).

Moreover, α-MSH was also reported to be a suppressor of proinflammatory non-cytokine regulators such as nitric oxide (a well-known common inflammatory mediator), prostaglandin E (PGE), and ROS. The ability of α-MSH in suppressing LPS- and cytokine-stimulated nitric oxide production and inducible nitric oxide synthase expression was first reported in murine macrophages expressing MC1R (65). Since then, similar findings were reported in cytotoxic agent-induced RAW 264.7 cells, THP-1 cells, human melanoma FM55 cells, murine microglial cells, rat PBMCs and astrocytes (134, 135, 140–143, 157, 158). It was demonstrated that α-MSH suppresses cytokine-stimulated PGE production in a cell-specific manner, with inhibitory effects observed in IL-1-induced fetal human lung fibroblasts and TNF-α-stimulated FM55 melanoma cells, but no effects observed in TNF-α-induced HaCaT keratinocytes (144, 159). In addition, α-MSH was recently reported to inhibit LPS- or phorbol ester-induced synthesis of superoxide radicals in rat neutrophils (145) and IL-8-stimulated oxidative burst in human monocytic cell line (132), suggesting an regulatory effect on the cellular redox balance and apoptotic pathways similar as that of a radical scavenger (160).

Other mechanisms that contribute to the anti-inflammatory actions of α-MSH have also been proposed. For instance, α-MSH was reported to induce the mRNA and protein levels of IL-10, a well-known potent cytokine suppressor, in human keratinocytes and monocytes, respectively (150, 161). In addition, the abilities of α-MSH in inducing CD25 and CD4 markers of regulatory T cells, suppressing the production IFN-γ of inflammatory T cells, and inhibiting bacterial antigen-induced proliferation of T lymphocytes were reported by several groups (161–163).

### **α**-MSH Effects *in vivo*

Similar to ACTH, α-MSH exhibits anti-inflammatory effects in a wide variety of animal models of experimental inflammation disorders. For instance, Glyn and Lipton first showed that α-MSH is able to inhibit temperature increase in rabbits with leukocytic pyrogen-induced fever (112). Since then, the antipyretic properties of α-MSH was confirmed in several animal models (such as leukocytic pyrogen-induced rabbits and guinea pigs, and bacterial endotoxin-treated squirrel monkey) with the peptide administrated centrally, peripherally, or intragastrically (164–170).

Following the therapeutic use of ACTH in patients with rheumatoid arthritis, the anti-inflammatory actions of α-MSH in experimental rheumatoid arthritis have been also well-explored. In 1994, Ceriani et al. showed that α-MSH, injected intraperitoneally twice daily, markedly inhibits the clinical and histological signs of adjuvant-induced arthritis (171). It should be noted that although α-MSH exerts similar anti-inflammatory actions on joint inflammation as prednisolone, the effectiveness of α-MSH and prednisolone on the weight regulation of experimental rats are different, with no weight change observed between α-MSH-treated and untreated animals but a significant weight loss in prednisolone-treated animals compared to the control animals (171).

In addition, α-MSH has also been shown to be a potent inhibitor of systemic inflammation, including sepsis syndrome, septic shock, acute respiratory distress syndrome (ARDS), and several other conditions. Martin and Lipton showed that intravenous injection of α-MSH reduces endotoxin-induced acute phase response in rabbits (172). Subsequently, similar results were reported in endotoxemic mice when the peptide was administrated centrally or peripherally (173). In addition, Catania and colleagues demonstrated that systemic injection of α-MSH or gentamicin increases survival rate in a mouse model of peritonitis/endotoxemia/septic shock (174). Interestingly, it achieves even greater survival rates when α-MSH and gentamicin were given in combination, suggesting the anti-inflammatory effects of α-MSH and gentamicin are independent and additive to each other (174). In endotoxin-induced ARDS rats characterized by massive neutrophil influx into the lung and damage to lung epithelium, systemic administration of α-MSH reduces leukocyte concentration in the bronchoalveolar lavage fluid (175). Similar salutary effects were observed in acute bleomycin-induced lung injury in rats and α-MSH was also shown to regulate stress-, inflammation-, and fluid homeostasis-related genes, which are known to be involved in the development of acute lung injury stress response (175).

### Mechanism of Anti-inflammatory Effects of **α**-MSH

With the aid of numerous cell culture systems, a variety of mechanisms as discussed above, including inhibition of nuclear transcription factor NF-κB activation, suppression of proinflammatory cytokine production and adhesion molecule expression, restraint of proinflammatory non-cytokine regulators, induction of cytokine suppressors, as well as modulation of lymphocyte function and proliferation, have been demonstrated to contribute to α-MSH-mediated anti-inflammatory effects, which are further validated in animal models of experimentally induced fever, rheumatoid arthritis, ARDS, inflammatory skin and bowel diseases, and brain inflammation (160).

Similar to ACTH, it is thought that the anti-inflammatory effects of α-MSH might be mediated through activation of specific MCRs expressed in peripheral immune cells and hypothalamic neurons. For instance, MC3R was shown to be required for ACTH- and α-MSH-mediated anti-inflammatory effects in rheumatoid arthritis (7). The α-MSH-induced inflammation-modulatory signaling pathways in the CNS are thought to be mediated by neural MC4R (176). MC1R and MC5R might be involved in α-MSH-mediated modulation of proinflammatory cytokines and collagens in human articular chondrocytes (68). Furthermore, protective effects of α-MSH in autoimmune uveoretinitis require a functional MC5R (177).

### Therapeutic Potential of **α**-MSH in Inflammatory Diseases

Current studies attempting to explore the therapeutic potential of α-MSH for treatment of inflammation disorders mainly focus on the C-terminal tripeptide of α-MSH, KPV, and its derivative KdPT, which retain the anti-inflammatory effect but abandon its pigmentary action. Indeed, KPV and KdPT, similar to α-MSH, at molecular levels, were also shown to regulate a wide spectrum of signaling pathways involved in inflammation-related processes, including suppression of NF-κB activation, TNF-α synthesis, and induction of IL-10 (160).

Furthermore, in animal models of experimentally induced fever, brain inflammation, skin inflammation, rheumatoid arthritis, and systemic inflammation, KPV or KdPT was reported to reduce leukocytic pyrogen- or IL-1-induced hyperthermia (170, 178), LPS-induced activation of NF-κB (128), picrylic acid-, TNF-α-, IL-1β-, or IL-6-induced ear swelling (179–181), γ-carrageenan-induced hind paw edema (182), MSU crystal- or IL-1β-induced peritonitis and neutrophil accumulation (139), and dextran sodium sulfate-induced colitis with inflammatory cell infiltration and myeloperoxidase activity (183, 184), indicating therapeutic potential of α-MSH for treatment of inflammatory diseases.

It should be noted that intravenously administered full-length α-MSH and its tripeptides can only last in circulation for a few minutes due to the presence of several serum proteases (185). For instance, neutral endopeptidase 24.11, one of the serum proteases expressed on the cell membrane of a variety of cell types, can cleave α-MSH and thereby directly limit its biological activity (186). Although the pharmacokinetics of KPV and KdPT in circulation are still not clear, it may be speculated that D-enantiomers of KPV, compared to their stereochemical analogs, are more resistant to proteolysis of peptidases (160). Therefore, it is of great importance to identify a better route of administration of these peptides depending on the specific inflammatory disease.

In terms of the safety profiles of α-MSH and its tripeptides, very few data are currently available due to lack of toxicity studies. However, the super-potent analog of α-MSH, [Nle^4^-D-Phe^7^]-MSH, when administrated intravenously at doses of up to 0.6 mg/kg, results in occasional gastrointestinal upset and facial flushing without major detrimental effects (187, 188). In this scenario, α-MSH and its tripeptides should have better safety profiles compared to traditional immunosuppressive therapies and biologics, known to cause liver and kidney injury, bone marrow suppression, gastrointestinal upset, hypertension, and dyslipidemia.

Interestingly, recent studies demonstrated antimicrobial activity of α-MSH and KPV in eliminating two representative pathogens, *Staphylococcus aureus* and *Candida albicans* (189). This is opposite to established immunosuppressive and anti-inflammatory therapies that usually enhance the risk for infection during the prolonged process. These advantages over the conventional immunosuppressive therapies highlight α-MSH and related-tripeptide as attractive candidates for treatment of human inflammatory diseases. However, compared to α-MSH, KPV, and KdPT might confer more advantages to be utilized as clinical therapy for the treatment of immune-mediated inflammatory disorders since they are easier to synthesize due to smaller size and lower cost, theoretically with no pigmentary effect, more resistant to bacterial infection, and more easily to be administrated locally for several inflammatory diseases (160).

## Other Melanocortins and Inflammation

Although β- and γ-MSHs share similar affinity for the immunomodulatory MC1R and MC3R as ACTH and α-MSH, the involvement of β- and γ-MSHs in the regulation of inflammation are less understood due to lack of experimental evidences.

The central antipyretic actions of γ_2_-MSH during the inflammatory response was reported by Bock et al., who showed that intraseptal or intraventricular infusion of γ_2_-MSH inhibits LPS-induced fever in guinea pig (190). Recently, the anti-inflammatory activity of γ_2_-MSH were further confirmed by Getting and colleagues, who demonstrated that in an *in vivo* murine model of peritonitis and *in vitro* cultured macrophages, γ_2_-MSH causes a dose-dependent attenuation of polymorphonuclear leukocyte migration (37). This inhibition was shown to be associated with a reduction in KC and IL-1β levels (37). In addition, SHU9119 blocks the ability of γ_2_-MSH to inhibit neutrophil migration, KC, and IL-1β release, suggesting effects of γ_2_-MSH are mediated by the MC3R or MC4R or both (37). However, only the mRNA and protein of MC3R, but not MC4R, were confirmed by RT-PCR and western blotting in murine and rat peritoneal macrophages (37). The MC3R is functional since it responds to γ_2_-MSH stimulation in initiating production of intracellular cAMP and this effect is blocked in the presence of SHU9119 (37). They concluded that γ_2_-MSH can inhibit the experimental inflammatory response by selectively activating MC3R on peritoneal macrophages.

Similar findings were observed in a recessive yellow mouse strain harboring a frameshift mutation in *Mc1r* gene that results in non-functional MC1R protein (191). Taking advantage of this mouse model and more selective melanocortin ligands, the same group showed that a functional MC1R is not necessary to elicit the anti-inflammatory actions of melanocortin peptides since (1) the recessive yellow mice does not display any signs of ongoing inflammatory response; (2) wild type and the recessive yellow mice showed no difference in MSU crystal-induced peritonitis as assessed by polymorphonuclear leukocyte migration and release of KC; (3) pre-administration of γ_2_-MSH reduces MSU crystal-induced polymorphonuclear leukocyte migration equally in wild-type and recessive yellow mice and this is associated with a reduction in IL-1β levels; (4) the selective MC1R agonist MS05 is inactive in inhibiting any of the inflammatory parameters in both wild type and recessive yellow mice (191).

Using male Sprague–Dawley rats, Xia et al. showed that intravenous infusion of γ_2_-MSH reverses LPS-induced hypotension and tachycardia, attenuates systemic inflammatory responses to endotoxin, prevents LPS-induced IL-1β gene expression in the brain and peripheral tissues, and inhibits LPS-induced increases in plasma nitric oxide levels, indicating a novel approach for modulating systemic inflammation through pharmacological manipulation of γ_2_-MSH-mediated autonomic activity (192). The specialized anti-inflammatory functions of β- and γ-MSHs within the brain were also reported by Muceniece and colleagues. In an acute mouse neuroinflammation model, four tested melanocortins were shown to reduce LPS-induced increases in brain nitric oxide as measured by electron paramagnetic resonance, with an order of effectiveness: β-MSH ≥ γ_1_-MSH = γ_2_-MSH > α-MSH (193).

## Conclusions

Recent years have witnessed progressively increased scientific interests in melanocortins and its receptors due to their wide expression and functions. A variety of *in vitro, in vivo*, and clinical studies have demonstrated that the anti-inflammatory effects of melanocortins are achieved in glucocorticoid-dependent (unique for ACTH) and -independent manners. Recent improvement in understanding the mechanisms by which melanocortins regulate immune responses have yielded renewed interest in RCI as a therapeutic option for several inflammatory diseases, since it may improve disease control, quality of life, and reduce healthcare utilization. In addition, the advantages of α-MSH-related tripeptides over the traditional melanocortins indicate that they could be utilized as novel therapeutics for the treatment of inflammatory disorders.

## Author Contributions

All authors listed have made a substantial, direct and intellectual contribution to the work, and approved it for publication.

### Conflict of Interest

WW, D-YG, and Y-JL, Employees of Xiamen Huli Guoyu Clinic, Co., Ltd., Xiamen, China The remaining author declares that the research was conducted in the absence of any commercial or financial relationships that could be construed as a potential conflict of interest.

## Abbreviations

ACTH: adrenocorticotropic hormone
AgRP: agouti-related peptide
ARDS: acute respiratory distress syndrome
CNS: central nervous system
HDMEC: human dermal microvascular endothelial cell
HMEC-1: human microvascular endothelial cell
ICAM-1: intercellular adhesion molecule-1
IFN-γ: interferon-γ
KC: keratinocyte-derived chemokine
MSH: melanocyte-stimulating hormone
OPC: oligodendroglia progenitor cell
PBMC: peripheral blood mononuclear cell
PC: prohormone convertase
PGE: prostaglandin E
POMC: pro-opiomelanocortin
MCR: melanocortin receptor
MSU: monosodium urate
RCI: repository corticotropin injection
ROS: reactive oxygen species
TNF-α: tumor necrosis factor-α.
